# An NMR Spectroscopic Investigation of Aluminosilicate Gel in Alkali-Activated Fly Ash in a CO_2_-Rich Environment

**DOI:** 10.3390/ma9050308

**Published:** 2016-04-26

**Authors:** Sol-Moi Park, Jeong-Gook Jang, Seen-Ae Chae, Haeng-Ki Lee

**Affiliations:** 1 Department of Civil and Environmental Engineering, Korea Advanced Institute of Science and Technology, 291 Daehak-ro, Yuseong-gu, Daejeon 34141, Korea; solmoi.park@kaist.ac.kr (S.-M.P.); jangjg@kaist.ac.kr (J.-G.J.); 2 Western Seoul Center, Korea Basic Science Institute, University-Industry Cooperation Building, 150 Bugahyeon-ro, Seodaemun-gu, Seoul 03759, Korea; saechae@kbsi.re.kr

**Keywords:** geopolymer, fly ash, carbonation, alkaline activation, NMR spectroscopy

## Abstract

The present study investigated aluminosilicate gel in alkali-activated fly ash exposed to a CO_2_-rich environment by means of NMR spectroscopy. The alkali-activated fly ash was exposed to an atmospheric CO_2_ concentration of 10% after curing at 80 °C initially for 24 h. Under high concentrations of CO_2_, highly reactive components Na and Al, which completely reacted within the first few hours, were unaffected by carbonation, while Si, with relatively slower reactivity, behaved differently. Despite a lower degree of the reaction in the carbonated sample, the monomeric silicates rapidly became of higher polymerization, meaning that exposure to high concentrations of CO_2_ caused Si to form a binding gel phase. Consequently, the carbonated sample possessed a higher amount of binding gel. The obtained results may be useful to understand the fundamental chemistry and behavior of aluminosilicate gel under high concentrations of CO_2_.

## 1. Introduction

The binding gel phase in alkali-activated fly ash (AFA), referred to as aluminosilicate gel, geopolymer gel (N-A-S-H) has been the topic of numerous studies in comparison with the main gel phase of Portland cement, calcium silicate hydrate (C-S-H), due mostly to the potential development of a construction material with a lower CO_2_ footprint and improved durability against aggressive conditions [1,2,3]. An enormous amount of knowledge on geopolymer chemistry has been gained owing to intensive research over the past two decades such that the roles of reactive components in the precursor [4,5,6,7] and the speciation of the binding gel phase [8] are well defined at present.

Recent studies are focused on the performance capabilities of the materials and stability of the binding gel under aggressive conditions, such as a CO_2_-rich environment [9]. A general consensus is that geopolymer gel is unharmed by carbonation [1], whereas the reaction path and properties of the material may be affected [10]. In particular, AFA exposed to a CO_2_-rich environment at an early age (after 24 h of initial curing) exhibited a significant decline in its structural integrity [1]. It was also reported that carbonation alters in the chemistry and pH of the pore solution in the gel [11], which in turn hinders further activation of the fly ash particles [10]. The major effect of carbonation on the Ca-free binder system is the modification of the alkali cations which initially charge-balance Al and become a carbonation product [9]. Despite the relatively few studies in this area, it is certain that the interaction between CO_2_ and the binding gel phase can only occur at an early age, at which point gel formation is incomplete.

It is therefore concluded that the time at which AFA is exposed to a CO_2_-rich environment has a detrimental effect on the phase evolution of the binder gel in AFA. The maturity of the gel and the reactivity of the components at which AFA is exposed to CO_2_ are closely associated with the development of strength. This indicates that curing AFA for a sufficient period of time may lead to higher strength development under high CO_2_ conditions, in contrast to that observed in the earlier work [1,11]. In a recent work conducted by the authors [12], AFA, which was exposed to an atmospheric CO_2_ concentration of 10%, exhibited a strength greater than the unexposed one. The increased strength of the carbonated AFA may have been caused by the decrease in the porosity due to the formation of nahcolite, but the FT-IR spectra clearly showed that the gel in the carbonated AFA was in a more mature state (higher Si/Al) [12]. Therefore, the binding gel phase in AFA is definitely modified to some extent under carbonation and thus requires a more detailed investigation. The present study adopted the NMR spectroscopic technique, which is particularly useful for the speciation of a particular nucleus in an amorphous material. In particular, ^29^Si, ^27^Al and ^23^Na NMR spectroscopy methods were used to probe the nuclei constituting the binding gel phase, and ^13^C NMR spectroscopy was used to examine the carbonation product in AFA exposed to a CO_2_-rich environment.

## 2. Materials and Methods

Class F fly ash was used as a geopolymer precursor. It was activated with an activating solution with a silicate modulus of 1.0 and a Na_2_O dosage of 6.5 g per 100 g of fly ash. The liquid to solid ratio was 0.5 by mass. The details regarding the sample preparation can be found in an earlier study [12]. All samples were initially cured in a chamber at 80 °C for 24 h. The following exposure condition was applied thereafter: (a) Samples were sealed to remove the effect of carbonation; or (b) samples were placed in an accelerated carbonation chamber to expose them to an atmospheric CO_2_ concentration of 10% for 28 days. A temperature of 20 °C and humidity of 65% were identically applied in both exposure conditions. For the designation of the samples, “uncarbonated samples” refer to the former, while “carbonated samples” refer to the latter throughout this paper.

The aluminosilicate gel in AFA under a CO_2_-rich environment was investigated by means of solid-state ^29^Si, ^27^Al, ^23^Na, and ^13^C magic-angle spinning (MAS) nuclear magnetic resonance (NMR) and liquid-state ^29^Si NMR spectroscopy. The surfaces of the specimens were sampled to ensure that the carbonated area was used in the analysis. Solid-state ^29^Si MAS NMR spectra were collected at 119.2 MHz using a HX-CPMAS probe and a 5-mm zirconia rotor with a Teflon spacer at a spinning speed of 10.0 kHz. A pulse width of 30° and 2.2 μs with a relaxation delay of 22 s were employed. The chemical shifts were referenced to tetrakis(trimethylsiyl)silane at −135.5 ppm with respect to TMS. Solid-state ^27^Al MAS NMR spectra were collected at 156.3 MHz using a HX-CPMAS probe and a 2.5-mm o.d. low-Al background zirconia rotor at a spinning speed of 22.0 kHz. A pulse width of 30° and 1.8 μs with a relaxation delay of 2 s were employed. The chemical shifts were referenced to AlCl_3_ at 0 ppm. Solid-state ^23^Na MAS NMR spectra were collected at 158.7 MHz using a HX-MAS probe and a 2.5-mm zirconia rotor at a spinning speed of 28.0 kHz. A pulse width of 80° and 1.3 μs with a relaxation delay of 10 s were employed. The chemical shifts were referenced to NaCl at 0 ppm. Solid-state ^13^C MAS NMR spectra were collected at 150.9 MHz using a ceramic module T3 HX-CPMAS probe and a 5-mm o.d. zirconia rotor with a boron nitride spacer at a spinning speed of 10.0 kHz. A pulse width of 35° and 2.0 μs with a relaxation delay of 15 s were employed. The chemical shifts were referenced to methyl of HMB at 17.3 ppm with respect to TMS. Liquid-state ^29^Si NMR spectra were collected at 99.3 MHz with ^1^H inverse gated decoupling using a SW-PFG probe and a 5-mm o.d. glass NMR tube. A pulse width of 30° and 2.2 μs was employed. The chemical shifts were referenced to TMS. The signal from the tube was subtracted from the spectra. All NMR experiments were conducted using a ^Unity^INOVA 600 NMR spectrometer (Varian Inc., Palo Alto, CA, USA) with a 14.1 Tesla wide-bore magnet and a ^Unity^INOVA 500 NMR spectrometer with an 11.7 Tesla narrow-bore magnet.

## 3. Results

### 3.1. Solid-State ^29^Si MAS NMR Spectroscopy

**Figure 1:**
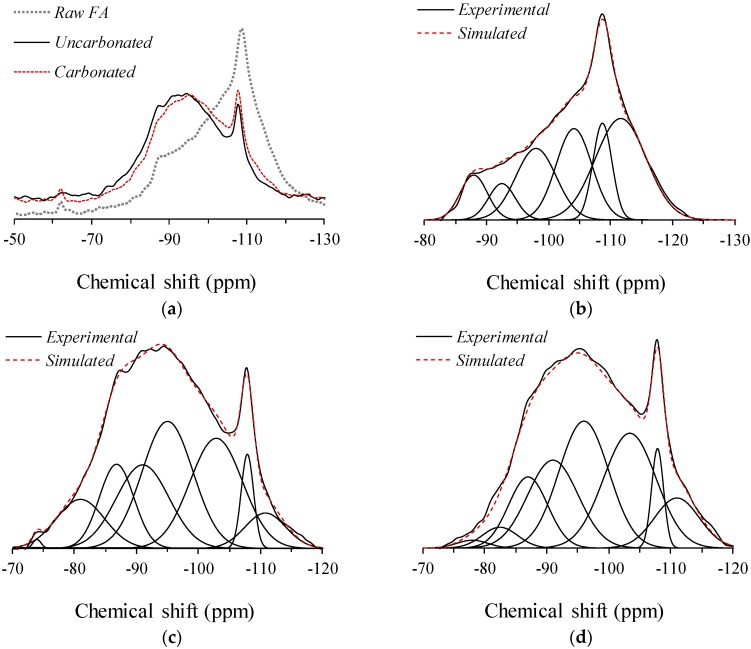
^29^Si MAS NMR spectra of (**a**) all; (**b**) raw fly ash; and (**c**) uncarbonated and (**d**) carbonated alkali-activated fly ash.

The ^29^Si MAS NMR spectra of the raw fly ash and the uncarbonated and carbonated AFA are shown in Figure 1. Each spectrum was deconvoluted into component peaks with the use of a Gaussian function by constraining the full width at half maximum (FWHM) to 10 ppm and with a minimum number of component peaks [1]. The peak position is in accordance with previous studies of AFA and geopolymers [8,13]. The simulation of component peaks was performed until the simulated fit converged to a chi-square value of 1E-12. The deconvolution result is provided in Table 1. It should be noted that the result provided in Table 2 is not an absolute value but only provides a point of comparison between the samples within the scope of this study. The resonances at −87 ppm, −92 ppm, −96 ppm, and −103 ppm in all spectra are assigned to Q^4^(*n*Al) sites, where *n* = 4, 3, 2, and 1, respectively [8], attributed to the presence of the glassy phase Si-O-Al in the raw fly ash and gel phase in the AFA. The resonances at −108 ppm and −111 ppm are associated with the Q^4^(0Al) site corresponding to the presence of crystalline Si (*i.e.*, quartz) and to the unreacted glassy phase Si-O-Si [6,14]. The resonance at −62 ppm, only observed in the spectra of the unreacted fly ash and carbonated AFA, is attributed to the signal from the Si-C linkage [15].

Upon alkaline activation, the intensity of the Q^4^(4Al), Q^4^(3Al), Q^4^(2Al), and Q^4^(1Al) increased, while that of the Q^4^(0Al) decreased, indicating the consumption of the glassy phases Si-O-Al and Si-O-Si and the formation of the binding gel phase. Meanwhile, the relative area corresponding to the amounts of residual silanol groups and unreacted fly ash, which was calculated from deconvolution, shows an interesting aspect. The resonance of the spectra at −80 ppm and −110 ppm shown in Figure 1a indicates that there were more residual silanol groups in the uncarbonated AFA, while there were more unreacted glassy phase Si-O-Si in the carbonated AFA. The phase composition derived from the deconvolution results is provided in Table 2. One can consider that these two phases are not the binding gel phase that provides strength. Upon carbonation, the degree of the reaction plainly declined, which is in good agreement with the findings of a previous study [10]. This was due to the modification of the alkali cations and the pH level in the pore solution [11]. Despite the lower degree of the reaction, the amount of aluminosilicate gel formed in the carbonated AFA was higher than that in the uncarbonated AFA; this is seemingly in fair agreement with the amount of residual silanol groups, far fewer in the carbonated AFA, thus requiring further investigation (see Section 3.5).

### 3.2. Solid-State ^27^Al MAS NMR Spectroscopy

**Figure 2:**
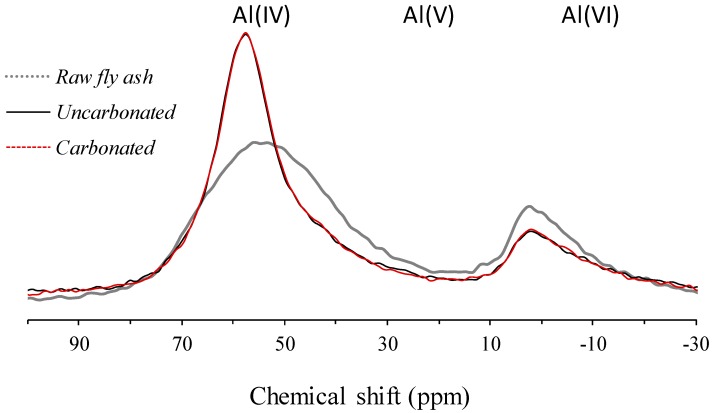
^27^Al MAS NMR spectra of raw fly ash and uncarbonated and carbonated alkali-activated fly ash.

^27^Al MAS NMR spectra of the raw fly ash and the uncarbonated and carbonated AFA are shown in Figure 2. The spectrum of the raw fly ash showed broad peaks corresponding to tetrahedral (70 ppm to 30 ppm) and octahedral (10 ppm to −30 ppm) Al sites [17]. The octahedral sites are attributed to the Al in the mullite and glassy phases, for which the Al environment is similar to that of mullite [18], whereas the tetrahedral sites are signals from the Al in the aluminosilicate glasses, where Al substitutes for Si [4,5]. Alkaline activation gave a rise to a sharp peak at 58 ppm, which is assigned to the q^4^ site and which reduces the intensity in the Al^VI^ environment, providing evidence for the formation of highly cross-linked geopolymer gel. Meanwhile, no significant change occurred in the Al environment of the AFA upon exposure to carbonation, as the spectra of the uncarbonated and carbonated samples were identical.

### 3.3. Solid-State ^23^Na MAS NMR Spectroscopy

**Figure 3:**
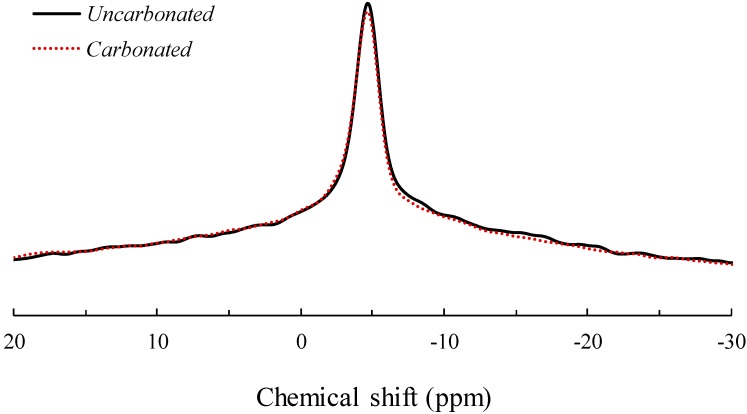
^23^Na MAS NMR spectra of uncarbonated and carbonated alkali-activated fly ash.

The ^23^Na MAS NMR spectra of the uncarbonated and carbonated AFA are shown in Figure 3. Typically, resonance toward lower chemical shifts indicates an increase in the Na-O bond length and the number of coordinates [19]. Both spectra showed resonance at −5 ppm, indicating that Na in both the uncarbonated and carbonated AFA was 5- to 6-coordinate. The obtained result was generally similar to that shown by ^27^Al MAS NMR spectroscopy, as the spectra were similar to each other. That is, the primary role of Na as a charge balancer for Al in AFA is unaltered by exposure to carbonation.

### 3.4. Solid-State ^13^C MAS NMR Spectroscopy

**Figure 4:**
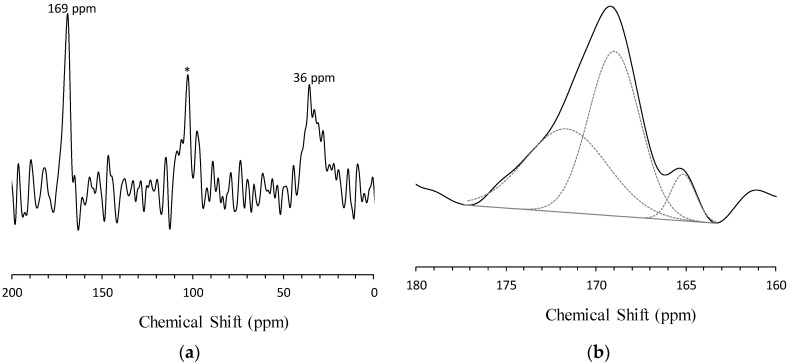
^13^C MAS NMR spectrum of carbonated alkali-activated fly ash. Spectral range of (**a**) 200 ppm to 0 ppm and; (**b**) 180 ppm to 160 ppm. The spinning sideband is marked with an asterisk.

The ^13^C MAS NMR spectrum of the carbonated AFA is shown in Figure 4. The carbonated sample contains peaks at 36 ppm and 169 ppm. The resonance at 36 ppm is attributed to the presence of carbon directly bonded to silicate [15]. This peak is attributed to the presence of silicon carbide, which was presumably formed during the sintering process and incorporated into the raw fly ash. The resonance at 169 ppm corresponds to the presence of carbonate groups, and its position is determined by the state of oxygen atoms; as this peak is asymmetric, it can be simulated into different components (Figure 4b) [20]. The component peak toward higher chemical shifts centered at 169 ppm is attributed to crystalline carbonate such as Na_2_CO_3_ or NaHCO_3_ [12], *i.e.*, an undistorted carbonate group [20]. Kohn *et al.* suggested that the component peaks at lower chemical shifts centered at 165 ppm correspond to distorted carbonate groups in the aluminosilicate framework [20]. In addition, the authors proposed two potential local environments of the latter carbonate groups in aluminosilicate glasses, which may describe the environments of the carbonate group in the AFA; one consists of two carbonate oxygens bridging C and Al or Si, and another one consists of one carbonate oxygen with a tri-coordinate structure [20]. However, further study is necessary to draw a correlation between the aluminosilicate distorted carbonate groups in terms of the geopolymer gel.

### 3.5. Si-Environment Seeded with CO_3_^2−^

**Figure 5:**
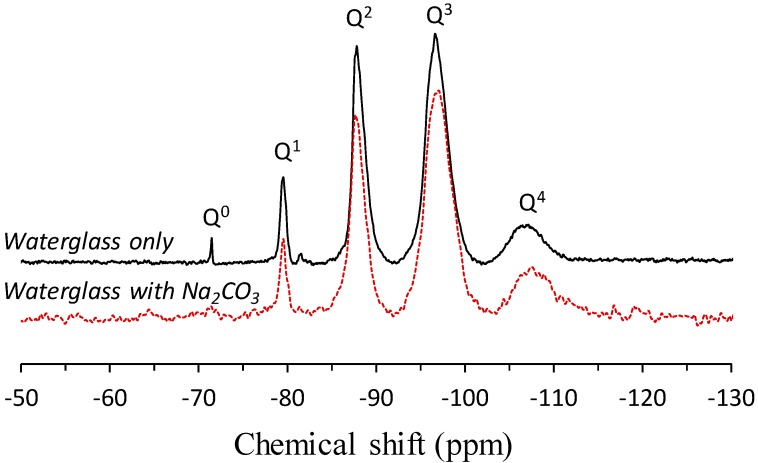
Liquid-state ^29^Si NMR spectrum of waterglass and solid-state ^29^Si MAS NMR spectrum of waterglass mixed with Na_2_CO_3_.

CO_3_^2−^ was seeded in an Al-free Si environment by preparing a mixture of waterglass (Korean Industrial Standards KS Grade-3, Korea; SiO_2_ = 29 wt %, Na_2_O = 10 wt %, and H_2_O = 61 wt %), in which Q^0^, Q^1^, Q^2^, Q^3^ and Q^4^ are present, and Na_2_CO_3_ (99.0% purity) powder to provide an elaborate investigation of the local Si structure under a CO_3_^2−^ saturated condition. The dissolution of Na_2_CO_3_ powder in the waterglass was assumed to release CO_3_^2−^, similar to the dissolution of carbon dioxide in the pore solution. The ^29^Si NMR spectra of the waterglass and that mixed with Na_2_CO_3_ are shown in Figure 5. It should be noted that liquid-state NMR spectroscopy was adopted to probe the local environments of Si present in the waterglass in an aqueous state. Similarly, solid-state NMR spectroscopy was adopted for the waterglass/Na_2_CO_3_ mixture as it solidified instantaneously upon mixing. The waterglass showed resonances at the Q^0^, Q^1^, Q^2^, Q^3^ and Q^4^ sites, while the Q^0^ site was not observed in the waterglass/Na_2_CO_3_ mixture.

## 4. Discussion and Conclusions

The obtained results provide new insight into the chemistry of aluminosilicate gel exposed to a CO_2_-rich environment at an early age, and explain the strength enhanced by exposure to a CO_2_-rich environment observed in a recent work conducted by the authors [12]. It should be noted that the compressive strength of AFA exposed to conditions identical to the present study can be found in [12]. Firstly, the main gel constituents Si and Al behaved differently, while Na was in close proximity to Al. In the present study, the role of the Al in aluminosilicate framework and that of Na in charge-balancing these Al were not altered by carbonation. However, it was found that the reactivity of Al plays a vital role in determining the strength development of AFA exposed to a CO_2_-rich environment. In previous studies which reported a decline in strength due to exposure to high concentrations of CO_2_, this may have been due to the exposure time with respect to the reactivity and the reaction degree of the Al components in the fly ash [1,10]. Unlike Si, the Al environment in AFA cured at 85 °C was found to be mostly unchanged after 8 h [21], indicating that the reaction of Al was completed within the 8 h. In contrast, it is likely that the Al in AFA cured at 40 °C in an earlier work [1] was not fully reacted within 24 h, during which time the sample was exposed to a CO_2_-rich environment. Therefore, the decline in strength was attributed to the lower amount of the binding gel phase in the carbonated AFA, as exposure to CO_2_-rich environment resulted in the modification of alkali cations, which should have been charge-balanced Al but instead formed sodium carbonate/bicarbonate.

Previous studies of thermodynamic modelling of the pore solution in alkali-activated binders exposed to a CO_2_-rich environment showed that the pH level in a pore solution significantly reduced [11]. Therefore, this effect correlates with a significant reduction in the reactivity of fly ash [10]. This indicates that the dissolution of Si and Al from fly ash particles is significantly inhibited soon after the sample is exposed to a CO_2_-rich environment. That is, the binding gel phase formed after exposure to a CO_2_-rich environment is only provided by species which had dissolved before the exposure. Earlier studies of the silicate chemistry revealed that decalcified silica gel is able to gain strength at a pH between 7 and 10 and in the presence of anions and cations by forming a three-dimensional network silica gel, which may increase the strength as well [22,23]. In fact, the mobility of Si in a solution is highly dependent on the pH, as evidenced by earlier findings [21], which showed that a higher pH level results in a higher degree of polymerization in a soluble silicate solution (note that the tested SiO_2_/Na_2_O modulus was 0 to 1.17). If the pH level is reduced further, due to the presence of CO_3_^2−^ as in the present study, monomeric silicate no longer persists. Consequently, the following summary of the effect of exposure to a CO_2_-rich environment on a Si-environment is offered.

- The dissolved silanol groups (monomeric silicates) in AFA exposed to a CO_2_-rich environment rapidly forms Si with a higher degree of polymerization, as shown in Table 2, which shows that the carbonated AFA had fewer residual silanol groups.
- Exposure to a CO_2_-rich environment lowered the pH level in the pore solution, as evidenced in previous studies [9]; therefore, the gel forming in AFA was promoted.
- These two factors may contribute to the formation of the binding gel phase in carbonated AFA and are responsible for the strength enhanced in the AFA exposed to a CO_2_-rich environment, observed in a recent study by the authors [10].

The outcome of the binding gel phase in AFA exposed to a CO_2_-rich environment is mostly dependent on the time at which the AFA is exposed to the CO_2_-rich environment and on the reaction kinetics of Al. The reactivity of Al may be significantly hindered by exposure to a CO_2_-rich environment due to the modification of alkali cations, which reduces the pH and the availability of charge balancers. In contrast, the Al which has become part of the aluminosilicate gel structure is unaffected by exposure to a CO_2_-rich environment. This study may give important implications on the chemistry of alkali-activated binders under aggressive conditions such as environments involving high concentrations of CO_2_.

## Figures and Tables

**Table 1:** Deconvolution results of ^29^Si MAS NMR spectra of raw fly ash, uncarbonated and carbonated alkali-activated fly ash. The estimated uncertainty in site percentages is ±1%.

Site Type	Silanol Groups		Q^4^(4Al)	Q^4^(3Al)	Q^4^(2Al)	Q^4^(1Al)	Q^4^(0Al)	
**Chemical Shift (ppm)**	**−75**	**−82**	**−87**	**−92**	**−96**	**−103**	**−108**	**−111**
Raw Fly Ash	–	–	7.9%	6.3%	18.9%	21.2%	12.0%	33.7%
Uncarbonated	0.3%	9.6%	11.8%	17.9%	26.2%	23.6%	4.8%	5.9%
Carbonated	1.0%	3.1%	11.6%	18.5%	26.4%	25.2%	4.9%	9.3%

**Table 2:** Phase composition of uncarbonated and carbonated alkali-activated fly ash as derived from deconvolution results.

Site Type	Aluminosilicate Gel ^1^	Si/Al ^2^	Unreacted Fly Ash ^3^	Residual Silanol Groups ^4^
Uncarbonated	79.5%	1.79	10.7%	9.9%
Carbonated	81.6%	1.81	14.2%	4.1%

^1^ The sum of relative area assigned to Q^4^(*n*Al), where *n* = 1, 2, 3 and 4, as obtained from the deconvolution of NMR spectra; contribution of mullite was neglected. ^2^ Calculated from the Engelhardth equation where *n* = 1, 2, 3 and 4 [16]. ^3^ The sum of relative intensity assigned to Q^4^(0Al); contribution of quartz was neglected. ^4^ The sum of relative intensity assigned to silanol groups. ^1,3,4^ The amount of respective phases gives a qualitative comparison between uncarbonated and carbonated samples and therefore does not represent the absolute content of each phase present in the sample.
